# Supplementary material for: An open-source framework for large-scale, flexible evaluation of biomedical text mining systems
Source: J Biomed Discov Collab. 2008 Jan 29;3:1. doi: 10.1186/1747-5333-3-1 (PMC2276192; doi:10.1186/1747-5333-3-1)
Supplement: Additional file 1 — Evaluation platform source code. The additional file contains the software evaluation framework discussed in this paper. The most current release can be downloaded from SourceForge.net [1]. See the accompanying README file for instructions on installation and use. Also included in the distribution is a collection of UIMA wrappers for some commonly used BioNLP tools and annotated corpora. [file 1747-5333-3-1-S1.zip › bionlp_uima_repository-1.0.1/docs/api/src-html/edu/uchsc/ccp/util/nlp/annotation/comparison/SharedStartOrEndSpanComparator.html]

```
001    package edu.uchsc.ccp.util.nlp.annotation.comparison;
002    
003    import java.util.List;
004    
005    import edu.uchsc.ccp.util.nlp.annotation.Span;
006    
007    /**
008     * 
009     * For "shared start or end" comparison, the spans are required to have the same start or end indexes in order to return 0. If they do not share the
010     * same span start or end, then if the span start for span1 is prior to the span start for span2, then -1 is returned, 1 otherwise.
011     * 
012     * @author williamb
013     * 
014     */
015    public class SharedStartOrEndSpanComparator extends SpanComparator {
016    
017        /**
018         * Compare two spans using the shares-span-start-or-end match criteria.
019         */
020        @Override
021        public int compare(Span span1, Span span2) {
022            return sharesSpanStartOrEnd(span1, span2);
023        }
024    
025        /**
026         * Compare two lists of spans using the shares-span-start-or-end match criteria.
027         * 
028         * @param spanList1
029         * @param spanList2
030         * @return
031         */
032        @Override
033        public int compare(List<Span> spanList1, List<Span> spanList2) {
034            return sharesSpanStartOrEnd(spanList1, spanList2);
035        }
036    
037    }
```
